# Increased aortic stiffness in adults with chronic indeterminate Chagas disease

**DOI:** 10.1371/journal.pone.0220689

**Published:** 2019-08-02

**Authors:** Filippo Valbusa, Andrea Angheben, Alessandro Mantovani, Verena Zerbato, Andrea Chiampan, Stefano Bonapace, Paola Rodari, Davide Agnoletti, Guido Arcaro, Cristiano Fava, Zeno Bisoffi, Giovanni Targher

**Affiliations:** 1 Division of Internal Medicine, IRCCS Sacro Cuore – Don Calabria Hospital, Negrar, Verona, Italy; 2 Department of Infectious and Tropical Diseases, IRCCS Sacro Cuore – Don Calabria Hospital, Negrar, Verona, Italy; 3 Section of Endocrinology, Diabetes and Metabolism, Department of Medicine, University and Azienda Ospedaliera Universitaria Integrata of Verona, Verona, Italy; 4 Division of Cardiology, IRCCS Sacro Cuore – Don Calabria Hospital, Negrar, Verona, Italy; 5 Section of General Medicine and Hypertension, Department of Medicine, University of Verona, Verona, Italy; 6 Department of Diagnostic and Public Health, University of Verona, Verona, Italy; Universita degli Studi di Roma La Sapienza, ITALY

## Abstract

An ever-increasing number of patients with chronic indeterminate Chagas disease are diagnosed with early vascular and cardiac abnormalities, as cardiovascular imaging becomes more sensitive. However, the currently available information on aortic stiffness (a prognostic marker for adverse cardiovascular outcomes) in these patients is scarce. In this study, we consecutively recruited 21 asymptomatic Bolivian adult patients with chronic indeterminate Chagas disease and 14 Bolivian adults, who were seronegative for *Trypanosoma cruzi* infection. No participants had a prior history of heart disease, hypertension, diabetes, chronic kidney disease or atrial fibrillation. Carotid-femoral pulse wave velocity (cf-PWV), carotid-radial PWV (cr-PWV), carotid intima-media thickness and conventional echocardiographic measurements were recorded in all participants. Patients with chronic indeterminate Chagas disease had significantly higher cf-PWV (7.9±1.3 *vs*. 6.4±1.1 m/s, *p* = 0.003) and greater HOMA-estimated insulin resistance than subjects without Chagas disease. The two groups did not significantly differ in terms of age, sex, smoking, adiposity measures, blood pressure, plasma lipids, fasting glucose levels as well as cr-PWV, carotid intima-media thickness measurements, left ventricular mass and function. Presence of chronic indeterminate Chagas disease was significantly associated with increasing cf-PWV values (β coefficient: 1.31, 95% coefficient interval 0.44 to 2.18, *p* = 0.005), even after adjustment for age, sex, heart rate, systolic blood pressure and insulin resistance. In conclusion, asymptomatic Bolivian adult patients with chronic indeterminate Chagas disease have an early and marked increase in aortic stiffness, as measured by cf-PWV, when compared to Bolivian adults who were seronegative for *Trypanosoma cruzi* infection.

## Introduction

Chagas disease is regarded as a neglected tropical disease, and is a zoonosis from the Latin America, which is caused by the protozoan parasite *Trypanosoma cruzi* [1]. An estimated 8 million to 10 million people are infected with this parasite, primarily in the Americas [1]. However, as a consequence of migrations from Latin America, a globalization of the disease is under way, and there are currently thousands of infected Latin Americans living in Europe [2–4]. The prevalence of *T*. *cruzi* infection in Latin American migrants, living in Europe and Italy, is estimated to be nearly 4–5%, with the highest prevalence among migrants from Bolivia [3,4].

Chagas disease remains an important cause of illness and premature death [1,5]. The clinical course of the disease usually comprises an acute phase (often asymptomatic) and a chronic phase. Decades after the acute phase, patients enter the chronic phase of the disease, which in up to ~30% of cases is typically characterized by cardiomyopathy (characterized by conduction-system defects, dilated cardiomyopathy and congestive heart failure) and gastrointestinal disease (megaesophagus, megacolon or both) [1,5]. Chagas cardiomyopathy is more common than gastrointestinal Chagas disease [1,5]. However, most infected persons remain for life in the indeterminate phase of chronic Chagas disease, which is characterized by subpatent parasitemia, seropositivity for *T*. *cruzi*, absence of clinical signs and symptoms of cardiac and digestive involvement, and normal chest radiography and electrocardiography [1,5]. To date, increasingly more patients with chronic indeterminate Chagas disease are diagnosed with subtle vascular and cardiac abnormalities (as diagnostic methods become more sensitive for detecting the cardiovascular involvement in these patients) [6], but the prognostic value of such abnormalities remains unclear.

Increased aortic stiffness is an early vessel alteration, which has been shown to predict the development of major adverse cardiovascular events and improve risk reclassification in individuals at intermediate cardiovascular risk [7–10]. The clinical practice guidelines for management of hypertension have proposed the measurement of carotid-femoral pulse wave velocity (cf-PWV) as a valid tool for non-invasively assessing arterial (aortic) stiffness and for predicting the development of adverse cardiovascular outcomes [11].

Experimental studies in animals reported the presence of vasculitis of the aorta and large elastic arteries in the acute phase of *T*. *cruzi* infection [1,5]. This supports the possibility that patients with Chagas disease, especially with its indeterminate form, would also have abnormal aortic stiffness. To our knowledge, the published information on measurement of aortic stiffness in patients with chronic indeterminate Chagas disease is scarce [12]. We believe this topic is clinically relevant, because cf-PWV measurement in patients with chronic indeterminate Chagas disease might help to identify those with early abnormalities of large artery distensibility and at higher risk of adverse cardiovascular outcomes. In addition, cf-PWV measurement in patients with chronic indeterminate Chagas disease might provide further insights into the putative pathophysiological mechanisms of vascular dysfunction induced by chronic *T*. *cruzi* infection, such the aggravating role of aortic stiffness in the long-term risk of developing chronic Chagas cardiomyopathy.

That said, given that Bolivian immigrants are at high risk of Chagas disease and represent one of the largest Latin American communities in Northern Italy today [4], we sought to examine whether Bolivian adult patients in the indeterminate phase of chronic Chagas disease have an increased aortic stiffness, as measured by cf-PWV, when compared to Bolivian adults, who were seronegative for *T*. *cruzi* infection.

## Materials and methods

### Subjects

For this exploratory, cross-sectional study, we consecutively recruited a sample of 21 Bolivian adult patients with chronic indeterminate Chagas disease, and 14 Bolivian adults with negative serology for *T*. *cruzi* infection, who attended the Center for Tropical Diseases of the “IRCCS Sacro Cuore–Don Calabria” Hospital of Negrar (Verona) for a screening of Chagas disease and other parasitic infections during a period of 18 months. All these Bolivian individuals lived in the same town (Bergamo) in Northern Italy, a non-endemic area for Chagas disease [13]. Subjects with a documented history of heart disease, hypertension, dyslipidemia, diabetes, chronic kidney disease or permanent atrial fibrillation were excluded from the study. None of them were taking any medications for hypertension, dyslipidemia, diabetes or known to interfere with PWV measurements. No participants had a previous history of treatment with any trypanocidal drug.

The local Ethics Committee (the IRB of the IRCCS Sacro Cuore–Don Calabria Hospital of Negrar) approved the study protocol. All participants gave their written informed consent for participation in the study.

### Clinical and laboratory data

Body mass index (BMI) was measured as kilograms divided by the square of height in meters. Waist circumference was measured at the midpoint between the lowest rib and the iliac crest. Blood pressure (BP) was measured with an automated sphygmomanometer (Dinamap-8100 monitor). We also calculated the pulse pressure (PP) using the following equation: PP = systolic BP–diastolic BP. Mean arterial pressure (MAP) was defined as follows: MAP = [diastolic BP + (PP)/3]. Information on smoking history was obtained from all patients via interviews during medical examinations.

Venous blood samples were collected in the morning after an overnight fast. Measurements of serum glucose, lipids, total homocysteine and other biochemical blood parameters were obtained using standard laboratory procedures at the central Laboratory of our hospital. Insulin levels were measured using a chemiluminescent immunoassay method (LIAISON, DiaSorin, Saluggia, Italy). Homeostasis model assessment score (HOMA-IR that includes both fasting glucose and insulin levels in its equation) was used for estimating insulin resistance. Glomerular filtration rate (e-GFR) was estimated using the Chronic Kidney Disease Epidemiology Collaboration (CKD-EPI) equation [14].

### Diagnosis of chronic Chagas disease

According to the World Health Organization recommendations [1,5], the diagnosis of chronic Chagas disease was based on the concordant results of at least two serological assays with different antigens. Serum samples were tested by using two commercially available enzyme-linked immunosorbent assays, one based on a native antigen (BioELISA Chagas III, BiosChile, Santiago, Chile) and the other on a recombinant antigen (BioELISA Chagas, Biokit, Lliça d′Almunt, Spain), respectively. These serological tests have a reported sensitivity and specificity close to 100%. Results were expressed as the index between the absorbance of the test serum and the threshold value. In our study, patients with seropositivity for *T*. *cruzi* in the absence of clinical signs and symptoms of cardiac and digestive involvement and normal resting electrocardiogram, chest radiography and barium X-rays were considered to have chronic indeterminate Chagas disease [1,5].

### Hemodynamic and carotid-artery intima-media thickness measurements

The PulsePen device (DiaTecne srl, Milan, Italy), a validated arterial tonometer, was used for measuring central BP, cf-PWV and carotid-radial PWV (cr-PWV) non-invasively [15]. Cf-PWV is considered as the “gold standard” measure of arterial stiffness [16]. PulsePen measurements were realized as two consecutive measurements in the carotid and femoral arteries, both synchronized by electrocardiogram. The pulse wave transit time was calculated as the difference between the time delay of the femoral pulse wave and the carotid pulse wave in relation to the R-wave of the electrocardiogram. PWV was calculated as the distance between the measurement sites (carotid-femoral x 0.8) divided by transit time delay. The procedure has been described in detail previously [17]. Central BP values were estimated from carotid pressure waveforms obtained by the PulsePen device. It has been demonstrated that central BP values and pulse wave analysis measured noninvasively at the common carotid artery represent a reliable estimate of the data measured at the level of the aorta by invasive methods [15]. Central BP values were obtained after calibration of the carotid BP waveforms with brachial mean and diastolic BP measured noninvasively, as stated above, simultaneously with tonometric measurements. The augmentation index (AIx), which is a measure of wave reflection depending on arterial stiffness, heart rate and peripheral resistance [18], was defined as the ratio of augmentation pressure to PP and was expressed as percentage, i.e., AIx = (augmentation pressure/PP) × 100. Heart rate was measured simultaneously with arterial stiffness measurements. All the aforementioned vascular measurements were performed by an experienced physician, who was blinded to the participants’ clinical details.

In each subject, we also measured the common carotid artery intima-media thickness using an ultrasonography (Vivid S6, GE Vingmed, Horten, Norway) with dedicated software for automatic calculation of intima-media thickness in order to eliminate the operator-dependent component of the measurement. Three measurements were detected for each projection (anterior, anterior-lateral and posterior) for both carotid arteries.

### Conventional echocardiography

A trans-thoracic echocardiographic Doppler evaluation (Vivid 7, GE Vingmed, Horten, Norway) was performed in all participants by an experienced cardiologist, who was blinded to the participants’ clinical details, for measuring left ventricular (LV) diameters, wall thickness and mass according to international standard criteria [19]. LV end-diastolic and end-systolic volumes and ejection fraction at rest were measured at the apical 4-chamber and 2-chamber views. Left atrial maximal volume was also measured at the end of LV systole from the apical 4-chamber and 2-chamber views (by modified Simpson rule). All measurements were indexed to body surface area when appropriate. Pulsed-wave Doppler was used to measure the trans-mitral peak early diastolic velocity (E), peak late diastolic velocity (A), E/A ratio, E/e’ ratio and E-wave deceleration time.

### Statistical analysis

Due to the exploratory, hypothesis-generating design of the study, we did not perform a priori sample size calculation. However, based on a single study available in the literature [12], which reported a not significant difference of -0.3 m/s in the means of cf-PWV between subjects with chronic indeterminate Chagas disease and control individuals, we estimated a posteriori sample size of 26 individuals (17 with chronic indeterminate Chagas disease and 9 control subjects) for a study with a confidence interval of 95%, a statistical power of 80%, a two-group size ratio of 0.5 and an inter-group difference of cf-PWV means of 0.75 m/s. Data are expressed as means±SD, medians (inter-quartile ranges, IQR) or proportions. The Fischer’s exact test for categorical variables, the unpaired Student’s *t*-test for normally distributed continuous variables, and the Mann-Whitney U test for non-normally distributed continuous variables were used to compare the differences in clinical, biochemical and hemodynamic characteristics between subjects with and without chronic indeterminate Chagas disease. The independent association between presence of chronic indeterminate Chagas disease and cf-PWV values (included as a continuous measure) was tested by using a linear regression analysis. In particular, we performed three forced-entry linear regression models: the first model was unadjusted (unadjusted model); the second model was adjusted for age, systolic BP and HOMA-estimated insulin resistance (adjusted model 1); and, finally, the third regression model was further adjusted for sex and heart rate (adjusted model 2). Covariates included in these multivariable linear regression models were selected as potential confounding factors based on their biological plausibility and/or significance in univariable analyses. A *p*-value <0.05 was considered to be statistically significant. Statistical analyses were performed using STATA software, version 14.2 (STATA, College Station, Texas, USA).

## Results

Table 1 shows the main clinical and biochemical characteristics of Bolivian adult individuals stratified by presence of chronic indeterminate Chagas disease. Patients with chronic indeterminate Chagas disease were more likely to be insulin resistant (as reflected by higher fasting insulin levels and greater HOMA-IR score) compared to subjects who were seronegative for *T*. *cruzi* infection. Conversely, age, sex, measures of adiposity (BMI and waist circumference), smoking history, heart rate, systolic and diastolic BP, PP, MAP, plasma lipids, fasting glucose, e-GFR_CKD-EPI_ and homocysteine concentrations did not significantly differ between the two groups.

**Table 1:** Main clinical and biochemical characteristics of asymptomatic Bolivian individuals with and without chronic indeterminate Chagas disease.

	Overall sample (*n* = 35)	With chronic indeterminate Chagas disease (*n* = 21)	Without chronic indeterminate Chagas disease (*n* = 14)	*P* value
Age (years)	42.6 ± 8.5	44.2 ± 8.2	41.2 ± 8.5	0.26
Sex (male/female)	10/25	5/16	5/9	0.44
BMI (kg/m^2^)	26.8 ± 3.4	27.0 ± 3.8	26.4 ±2.6	0.89
Waist circumference (cm)	89 ± 10	89 ± 10	88 ± 11	0.70
Current smokers (%)	11.4	9.5	14.3	0.46
Heart rate (bpm)	62 ± 7	62 ± 7	63 ± 6	0.62
Systolic BP (mmHg)	118 ± 11	118 ± 11	116 ± 10	0.50
Diastolic BP (mmHg)	70 ± 9	69 ± 9	71 ± 9	0.70
PP (mmHg)	48 ± 9	49 ± 9	46 ± 8	0.13
MAP (mmHg)	86 ± 8	86 ± 9	86 ± 8	0.85
Total cholesterol (mmol/l)	4.9 ± 1.0	4.8 ± 1.1	5.1 ± 0.9	0.53
HDL cholesterol (mmol/l)	1.3 ± 0.3	1.3 ± 0.3	1.4 ± 0.4	0.18
LDL cholesterol (mmol/l)	2.8 ± 0.9	2.7 ± 0.7	3.0 ± 1.1	0.31
Triglycerides (mmol/l)	1.2 (0.8–1.8)	1.2 (0.8–1.9)	0.9 (0.7–1.8)	0.48
Fasting glucose (mmol/l)	5.2 ± 0.6	5.1 ± 0.6	5.4 ± 0.6	0.18
Fasting insulin (mIU/l)	6.0 (3.8–8.4)	6.7 (5.2–8.7)	4.2 (3.0–5.8)	0.01
HOMA-IR score	1.3 (0.9–2.0)	1.6 (1.1–2.1)	1.0 (0.7–1.4)	0.022
Creatinine (umol/l)	60 ± 13	58 ± 14	63 ± 11	0.23
e-GFR_CKD-EPI_ (ml/min/1.73 m^2^)	118 ± 12	118 ± 13	116 ± 10	0.92
Total homocysteine (umol/l)	8.6 (7.0–10.1)	8.6 (7.2–10.2)	8.5 (6.9–10.1)	0.73

Data are expressed as means ± SD, medians (IQR) or proportions. Differences between the two groups were tested by the Fischer’s exact test for categorical variables, the unpaired Student’s *t*-test for normally distributed continuous variables, and the Mann-Whitney U test for non-normally distributed continuous variables.

*Abbreviations*: BMI: body mass index; BP, blood pressure; e-GFR: estimated glomerular filtration rate (by using the CKD-EPI equation); HOMA-IR: homeostasis model assessment-insulin resistance; MAP, mean arterial pressure; PP, pulse pressure.

The vascular, hemodynamic and echocardiographic characteristics of participants are summarized in Table 2. Patients with chronic indeterminate Chagas disease had significantly higher values of cf-PWV and a greater prevalence of individuals with cf-PWV >10 m/s (i.e., a widely used cut-off for distinguishing individuals with abnormal aortic stiffness) compared with subjects without chronic indeterminate Chagas disease. In contrast, central BP values, augmentation index, cr-PWV, carotid artery intima-media thickness, trans-mitral E/A ratio, E/e’ ratio, Dte, LV and left atrial dimensions and volumes, LV mass index and LV ejection fraction did not significantly differ between two groups. Moreover, no participants had an E/e’ ratio >14 or other echocardiographic features of LV diastolic dysfunction.

**Table 2:** Vascular, hemodynamic and cardiac features of asymptomatic Bolivian individuals with and without chronic indeterminate Chagas disease.

	Overall sample (*n* = 35)	With chronic indeterminate Chagas disease (*n* = 21)	Without chronic indeterminate Chagas disease (*n* = 14)	*P* value
cf-PWV (m/s)	7.3 ± 1.4	7.9 ± 1.3	6.4 ± 1.1	0.003
cf-PWV >10 m/s (%)	28.6	42.9	7.1	0.028
cr-PWV (m/s)	9.7 ± 1.5	10.0 ± 1.7	9.2 ± 1.1	0.27
Augmentation index (%)	22 (9–35)	22 (11–33)	22 (2–42)	0.35
Central systolic BP (mmHg)	106 ± 10	106 ± 10	105 ± 10	0.39
Central PP (mmHg)	36 ± 7	38 ± 7	34 ± 7	0.12
Right common carotid IMT (mm)	0.57 ± 0.09	0.57 ± 0.08	0.56 ± 0.11	0.88
Left common carotid IMT (mm)	0.57 ± 0.10	0.56 ± 0.08	0.59 ± 0.13	0.41
LV end-diastolic volume index (ml/m^2^)	48.6 ± 8.0	51.6 ± 7.4	46.1 ± 7.8	0.07
LV end-systolic volume index (ml/m^2^)	15.9 ± 3.4	15.3 ± 2.7	16.6 ± 4.0	0.48
Left atrial volume index (ml/m^2^)	24.5 (20–31.5)	22.0 (17.5–28.3)	27.0 (20.7–33.5)	0.07
LV mass index (g/m^2^)	85.1 (65–105)	83.0 (65–101)	85.2 (63–107)	0.97
LV ejection fraction (%)	66 ± 5	66 ± 4	66 ± 5	0.76
E/e’ ratio	8.0 (7.0–9.1)	7.8 (6.5–8.8)	8.0 (7.9–10)	0.20
E/A ratio	1.3 ± 0.4	1.2 ± 0.3	1.5 ± 0.4	0.06
Dte (ms)	221 ± 37	217 ± 40	228 ± 34	0.43

Sample size, n = 35. Data are expressed as means ± SD, medians (IQR) or percentages. Differences between the two groups were tested by the Fischer’s exact test for categorical variables, the unpaired Student’s *t*-test for normally distributed continuous variables, and the Mann-Whitney U test for non-normally distributed continuous variables.

*Abbreviations*: BP, blood pressure; Dte: E-wave deceleration time; IMT: intima-media thickness; LV: left ventricular; cf-PWV, carotid-femoral pulse wave velocity; cr-PWV, carotid-radial pulse wave velocity; PP, pulse pressure.

**Fig 1:**
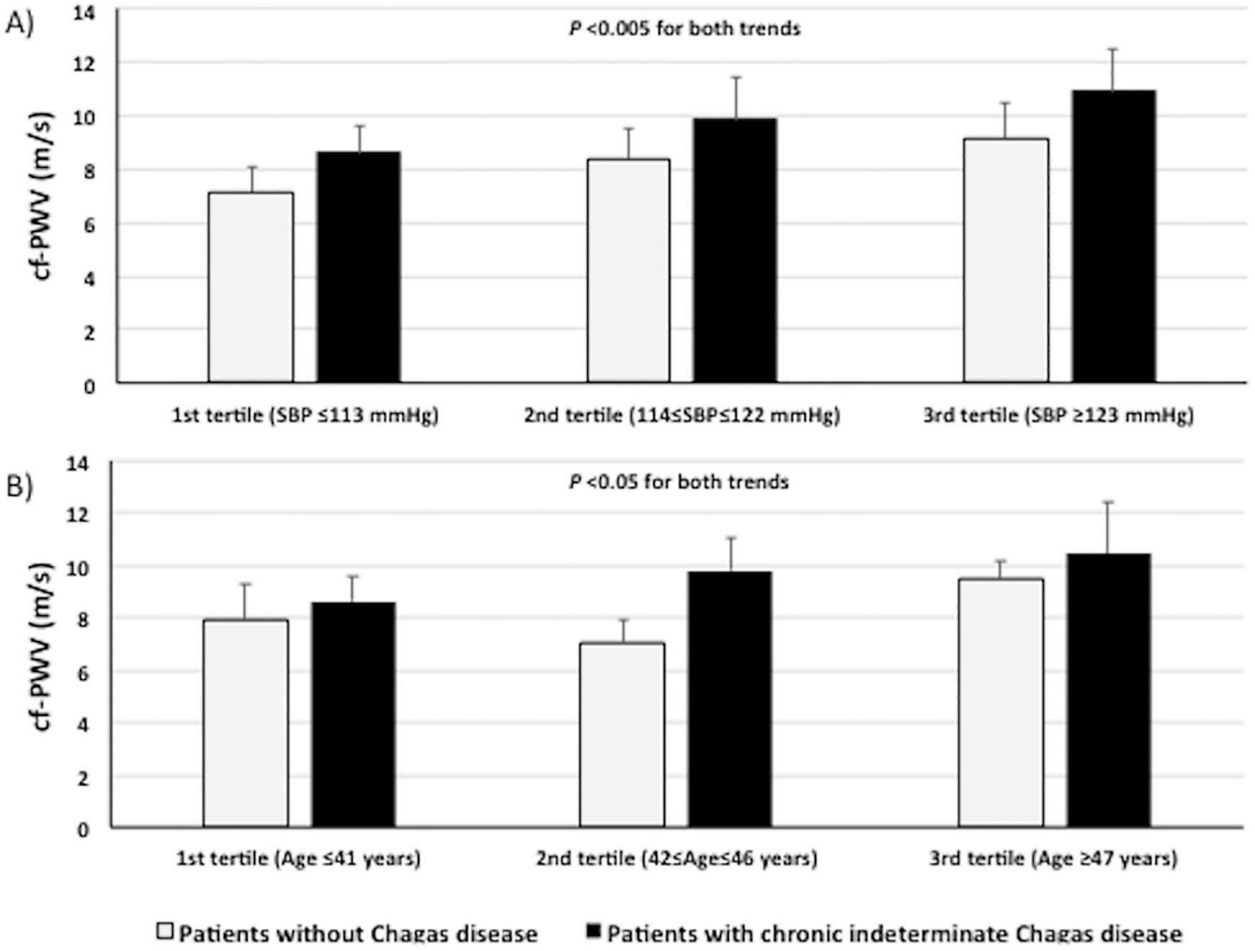
Mean ± SD values of carotid-femoral pulse wave velocity (cf-PWV) in asymptomatic Bolivian adults with and without chronic indeterminate Chagas disease, stratified either by systolic BP tertiles (panel A) or by age tertiles (panel B).

Fig 1 shows the mean values of cf-PWV among subjects with and without chronic indeterminate Chagas disease, stratified by tertiles of either systolic BP (panel A) or age (panel B), respectively. Cf-PWV values increased progressively across tertiles of systolic BP and age in both groups (*p*<0.05–0.005 for both trends). Notably, patients with chronic indeterminate Chagas disease had higher cf-PWV values in each tertile of both systolic BP and age compared with those without chronic indeterminate Chagas disease.

Table 3 shows the association between the presence of chronic indeterminate Chagas disease and cf-PWV (included as a continuous measure). In univariable linear regression analysis, the presence of chronic indeterminate Chagas disease was significantly associated with increasing cf-PWV values. The association between the presence of chronic indeterminate Chagas disease and cf-PWV remained statistically significant after adjustment for age, systolic BP and HOMA-estimated insulin resistance (adjusted model 1). Further adjustment for sex and heart rate did not weaken the significance of this association (adjusted model 2). Although we included five covariates in the adjusted model 2, we believe that the results of these two multivariable regression models exclude potential data overfitting. As expected, other variables that were independently associated with increasing cf-PWV values were older age and higher systolic BP. Notably, the adjusted model 2 explained ~68% of the total variability of cf-PWV (R^2^ model = 0.677).

**Table 3:** Association between presence of chronic indeterminate Chagas disease and carotid-femoral pulse wave velocity (cf-PWV) in asymptomatic Bolivian adults.

	*B* coefficient	95% Confidence Interval	Standardized *B* coefficient	*P* value
**Unadjusted model**				
Indeterminate Chagas disease (yes *vs*. no)	1.78	0.71 to 2.87	0.51	0.002
**Adjusted model 1**				
Indeterminate Chagas disease (yes *vs*. no)	1.37	0.47 to 2.27	0.39	0.004
Age (years)	0.06	0.01 to 0.11	0.30	0.02
Systolic BP (mmHg)	0.07	0.03 to 0.11	0.43	0.001
HOMA-IR score	0.10	-0.45 to +0.64	0.04	0.73
*Overall R*^*2*^ *adjusted model* = 0.621				
**Adjusted model 2**				
Indeterminate Chagas disease (yes *vs*. no)	1.31	0.44 to 2.18	0.38	0.005
Age (years)	0.06	0.01 to 0.12	0.31	0.02
Systolic BP (mmHg)	0.07	0.03 to 0.11	0.46	0.001
HOMA-IR score	0.23	-0.31 to +0.77	0.11	0.40
Sex (male *vs*. female)	-0.66	-1.66 to +0.34	-0.17	0.20
Heart rate (bpm)	-0.03	-0.10 to +0.03	-0.13	0.32
*Overall R*^*2*^ *adjusted model* = 0.677				

Sample size, *n* = 35. Data are expressed as beta (*B*) coefficients and 95% confidence intervals as tested by linear regression analysis. The dependent variable was cf-PWV (included as a continuous measure) for both unadjusted and adjusted linear regression models.

## Discussion

The main finding of our pilot study was that asymptomatic Bolivian adult patients with chronic indeterminate Chagas disease (without established cardiac disease, diabetes, hypertension, dyslipidemia, chronic kidney disease and atrial fibrillation and without a previous history of treatment with any trypanocidal drug) had remarkably higher values of cf-PWV, a greater prevalence of those with cf-PWV >10 m/s and greater insulin resistance compared with Bolivian adults, who were seronegative for *T*. *cruzi* infection. Conversely, age, sex, adiposity measures, plasma lipids, blood pressure, smoking, carotid artery intima-media thickness, cr-PWV, LV systolic/diastolic function and LV mass index were not significantly different between the two groups of subjects. Notably, the presence of chronic indeterminate Chagas disease remained significantly associated with increasing cf-PWV values (along with older age and higher systolic BP), even after adjustment for age, sex, heart rate, systolic BP and HOMA-estimated insulin resistance.

At first glance, our findings appear to be in contrast with the results reported by the only previously published study that examined the large artery distensibility, by means of PWV analysis, in chronic Chagas disease. Indeed, in a Brazilian case-control study, Villacorta *et al*. [12] reported that cf-PWV values (measured using the Complior device) were essentially superimposable among patients with chronic indeterminate Chagas disease (n = 16), patients with chronic Chagas disease and cardiac involvement [with either cardiac conduction defects (n = 18) or overt heart failure (n = 19)] and control subjects (n = 31) (cf-PWV: 8.4±1.1 *vs*. 8.2±1.5 *vs*. 8.2±1.4 *vs*. 8.7±1.6 m/s, *p* = 0.60, respectively). However, a reasonable explanation for the discrepancy with our data is that the lack of significant differences in cf-PWV values among the aforementioned four patient groups was likely due to the fact that most patients with chronic Chagas disease were treated with digoxin, diuretics or vasodilator drugs [12]. Therefore, it is plausible to assume that the use of these drugs may have affected cf-PWV measurements, leading to a "pseudo-normalization" of abnormal cf-PWV in these Chagas disease patients.

The lack of significant differences in cr-PWV values (i.e., a marker of peripheral muscular arterial stiffness) we observed between subjects with and without chronic indeterminate Chagas disease might appear an unexpected finding. However, it should be noted that the regional functional measurement of PWV provides only an average of PWV measurements over a confined segment of arteries that might have different biomechanical characteristics. Hence, the regional measurement of PWV might partly mask the early changes in viscoelastic and biomechanical properties of a specific arterial segment. Alternatively, it is also possible to hypothesize that due to a specific *T*. *cruzi* tissue tropism, the aorta and large elastic arteries might be affected earlier than small peripheral arteries in patients with chronic indeterminate Chagas disease. This has been experimentally documented in animal models where *T*. *cruzi*-infected CBA/J mice exhibited a marked vasculitis of the aorta, with significant infiltration of inflammatory cells into the adventitial layer (including CD4+, CD8+ T cells and macrophages) compared with non-infected mice of identical age [20]. A similar behaviour has also been reported for *Treponema pallidum* that has a specific tropism for the ascending aorta, causing a chronic inflammatory infiltrate of the medial and adventitial vasa vasorum, which ultimately leads to aortic aneurism during the tertiary syphilis [21]. On the other hand, adipose tissue is another important target tissue of *T*. *cruzi* and the infection of this tissue by the parasite is associated with an adverse impact on systemic metabolism, causing whole-body insulin resistance and low-grade, chronic inflammation that may further contribute to the stiffening of large elastic arteries [22,23]. Interestingly, in our study the significant differences in cf-PWV between subjects with and without chronic indeterminate Chagas disease persisted even after adjusting for HOMA-estimated insulin resistance. Unfortunately, in this study we did not measure plasma C-reactive protein, tumour necrosis factor-alpha or other inflammatory biomarkers that may actively contribute to the stiffening of the aorta and large elastic arteries.

Cardiac imaging is crucial to detect the cardiac involvement in patients with Chagas disease, stage the disease and stratify patient risk and address management [24]. Therefore, we believe that the present findings are clinically relevant, because they suggest that measurement of cf-PWV may identify Chagas disease patients with an early systemic vascular damage, and those who are also at higher risk of adverse cardiovascular outcomes. Interestingly, we found that subjects with chronic indeterminate Chagas disease had a mean cf-PWV of 7.9 m/s, whereas those without chronic indeterminate Chagas disease had a mean cf-PWV of 6.4 m/s; this difference in cf-PWV can be translated into an additional ~15 years of ageing, according to normal and reference values for PWV measurements based on a large European population [25]. In addition, we believe that our findings may also provide further insights into the putative pathophysiological mechanisms of vascular dysfunction in the chronic indeterminate phase of *T*. *cruzi* infection, suggesting that aortic stiffness might actively contribute to the development of chronic Chagas cardiomyopathy over time. However, future studies are needed to better understand whether cf-PWV measurement provides an earlier diagnosis of cardiac involvement in patients with chronic indeterminate Chagas disease.

To date, the pathophysiological mechanisms underpinning the association between chronic indeterminate Chagas disease and stiffening of the aorta and large elastic arteries are poorly understood. However, there is a growing consensus that parasite persistence is central to the disease, and that the inflammatory immune response of the host is the most important determinant for the establishment and progression of vascular damage, with *T*. *cruzi* strain virulence and tissue tropism as possible contributory factors [1,5,26–28]. Therefore, it is plausible that in the presence of a low-grade, chronic inflammatory state, the medial and adventitial extracellular matrix undergoes remodeling with increased collagen deposition, reduced elastin content, and increased infiltration of inflammatory cells. Experimentally, it has also been proposed a key role of macrophages, mitochondrial dysfunction and antioxidant/oxidant imbalance in the pathogenesis of chronic Chagas disease [29]. In addition, various animal models showed that T. cruzi invades endothelial cells and may cause nitric oxide synthase (eNOS)-derived NO-dependent vascular relaxation, and increased vascular contractility accompanied by augmented superoxide anions production [26]. All these processes may promote increased arterial stiffness, reduced elasticity, impaired distensibility, increased vasomotor tone and then increased aortic vascular fibrosis [16].

Our study has some important limitations that should be mentioned. First, the sample size of our exploratory study was relatively small (partly due to the difficulty of finding enough cases and controls among Bolivian migrants in Italy) and, thereby, the interpretation of our findings requires some caution. Second, since we included Bolivian individuals (living in the same area in Northern Italy), our results might not be necessarily generalizable to other Latin American migrants. Finally, although our subjects with and without chronic indeterminate Chagas disease were well comparable for age, sex, blood pressure and other established cardiovascular risk factors and we also adjusted the results for multiple potential confounding variables, residual confounding cannot be ruled out.

Despite these limitations, our study has also important strengths, including the completeness of the dataset, the use of validated methods for the non-invasive assessment of arterial stiffness, and the exclusion of Chagas disease patients with important comorbidities (overt cardiac complications, hypertension, diabetes, chronic kidney disease or atrial fibrillation) and those treated with trypanocidal agents or drugs known to interfere with vascular distensibility and vasodilation. We believe that including patients with such comorbidities or those treated with such medications might have confounded the interpretation of data.

In conclusion, our study shows that asymptomatic Bolivian adult patients with chronic indeterminate Chagas disease had significantly higher aortic stiffness, as measured by cf-PWV, in comparison with Bolivian adult individuals, who were seronegative for *T*. *cruzi* infection. Notably, this difference in cf-PWV remained significant even after adjustment for age, sex, heart rate, systolic BP and HOMA-estimated insulin resistance. Our finding suggests an early involvement of the aorta and elastic large arteries in patients with chronic indeterminate Chagas disease that might help to identify not only individuals at higher risk of major adverse cardiovascular outcomes, but might also suggest a possible contributing role of aortic stiffness in the development of chronic Chagas cardiomyopathy over time. However, larger follow-up and mechanistic studies are needed to better understand the link between chronic indeterminate Chagas disease and increased aortic stiffness, and to assess whether the early vascular damage observed during the indeterminate phase of the Chagas disease is an inactive or progressive stage.

## Supporting information

S1 Checklist STROBE Checklist. (DOCX)
